# Tetra­ammonium bis­(metforminium) di-μ_6_-oxido-tetra-μ_3_-oxido-tetra­deca-μ_2_-oxido-octa­oxidodeca­vanadium(V) hexa­hydrate

**DOI:** 10.1107/S2414314621006349

**Published:** 2021-06-25

**Authors:** J. Alberto Polito-Lucas, José A. Núñez-Ávila, Sylvain Bernès, Aarón Pérez-Benítez

**Affiliations:** a Instituto de Física, Benemérita Universidad Autónoma de Puebla, Av. San Claudio y 18 Sur, 72570 Puebla, Pue., Mexico; b Facultad de Ciencias Químicas, Benemérita Universidad Autónoma de Puebla, Av. San Claudio y 18 Sur, 72570 Puebla, Pue., Mexico; University Koblenz-Landau, Germany

**Keywords:** crystal structure, metformin, deca­vanadate, hydrogen bond

## Abstract

The title compound, (NH_4_)_4_(C_4_H_12_N_5_)_2_[V_10_O_28_]·6H_2_O, crystallizes with the deca­vanadate anion placed on an inversion centre in space group *P*  . This anion is surrounded by a first shell of ammonium cations and water mol­ecules, forming efficient N—H⋯O and O—H⋯O hydrogen bonds. A second shell includes metforminium monocations with a twisted geometry, also forming numerous inter­molecular hydrogen bonds. The complex three-dimensional network of non-covalent inter­actions affords a crystal structure in which the cations and anions are densely packed.

## Abstract 2

In the title salt, all O—H and N—H groups in the cations (metforminium and ammonium) and lattice water mol­ecules are donor groups for hydrogen bonds, giving a highly compact crystal structure.

## Graphical abstract

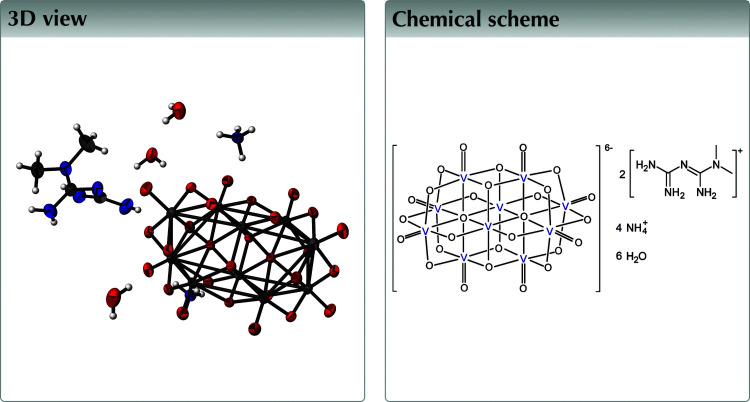

## Structure description

**Figure 1:**
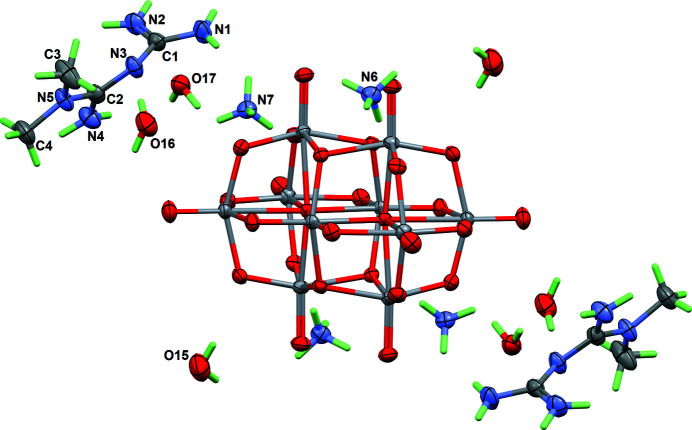
The mol­ecular entities in the structure of the title compound, with displacement ellipsoids for non-H atoms at the 50% probability level. Cations and water mol­ecules in the asymmetric unit are labelled.

Metformin hydro­chloride (Metf·HCl: 1,1-di­methyl­biguanide hydro­chloride; Niranjana Devi *et al.*, 2017 ▸) is the first-line therapy for type 2 diabetes. On the other hand, some anionic or cationic vanadium species, such as vanadate and vanadyl, have also been shown to be useful in the treatment of human diabetes (Domingo & Gómez, 2016 ▸). Based on this background, several groups belonging to the Autonomous University of Puebla are involved in the synthesis of compounds including both metformin and oxidovanadate derivatives, with the hope of achieving synergistic effects (Sánchez-Lombardo *et al.*, 2014 ▸). The associated chemical crystallography is rather complex, because due to its basic character metformin can be found in various states of protonation (neutral, cationic or dicationic forms), while the degree of condensation for the vanadate moiety strongly depends on the pH of the reaction medium. Finally, most of these compounds are crystallized with a number of water mol­ecules, which is unpredictable. The compound reported here includes one (V_10_O_28_)^6−^ anion, four ammonium cations, two metforminium(1+) cations HMetf^+^, and six water mol­ecules (Fig. 1 ▸).

The (V_10_O_28_)^6−^ anion is situated on an inversion centre in space group *P* 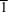 , and approaches the expected *D*_2*h*_ symmetry, which has been extensively reported (Bošnjaković-Pavlović *et al.*, 2011 ▸). The negative charges are balanced by four NH_4_^+^ and two HMetf^+^ cations. The high resolution of the measured diffraction data (*d*_min_ = 0.56 Å) unequivocally establishes that there is no protonation of the deca­vanadate. The HMetf^+^ monocation has its charge located mainly on N2. Furthermore, this cation is characterized by a dihedral angle of 54.85 (5)° between planes C2–C4/N3–N5 and C1/N1–N3. This twisted geometry is observed in several other compounds of metforminium(1+). Indeed, metformin and its cations HMetf^+^ and H_2_Metf^2+^ are highly flexible entities: the twist angle for 93 structures recovered from the CSD (Groom *et al.*, 2016 ▸) varies from 1 to 85°.

**Figure 2:**
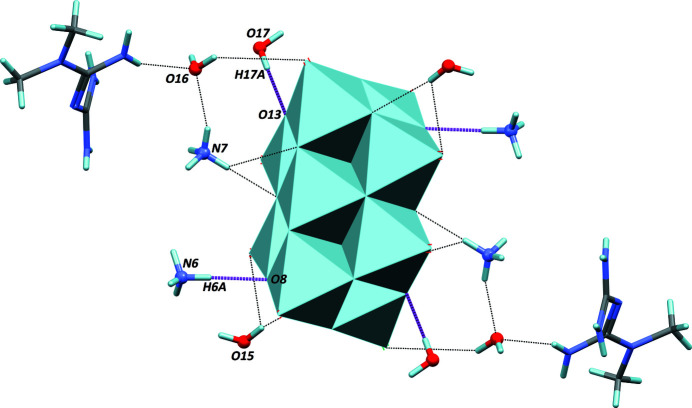
Main inter­actions between the (V_10_O_28_)^6−^ anion (polyhedral representation) and the cations and water mol­ecules. The strongest hydrogen bonds are represented as magenta dashed bonds (entries 7 and 19 in Table 1 ▸), while secondary hydrogen bonds are represented with thin black dashed lines.

**Figure 3:**
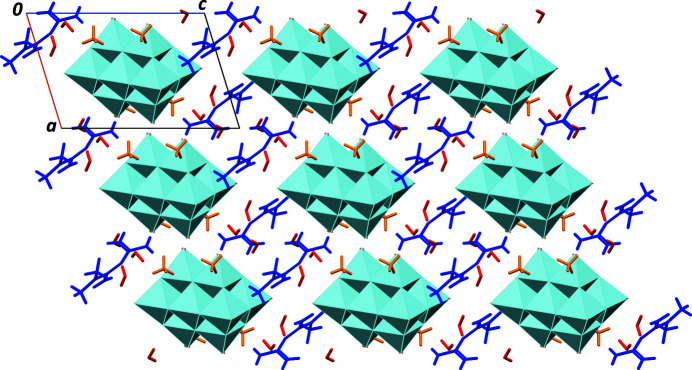
Part of the crystal structure of the title salt, viewed along [010]. Colour code: pale-blue polyhedra: (V_10_O_28_)^6−^ anions; orange: ammonium; blue: metforminium(1+); red: water.

In the crystal structure, anions and cations are well distributed, in such a way that the repulsive Coulombic forces between the highly charged anions are minimized. The deca­vanadate anion, the cations, and the crystal water mol­ecules engage in an extensive network of hydrogen bonds (Table 1 ▸). All N—H and O—H groups present in the asymmetric unit serve as donor groups. The two strongest hydrogen bonds are formed between the anion and one ammonium [N6—H6*A*⋯O8^v^; symmetry code: (v) −*x* + 1, −*y* + 2, −*z* + 1], as well as between the anion and a water mol­ecule (O17—H17*A*⋯O13; Fig. 2 ▸). As a consequence of the large number of hydrogen bonds, ions and mol­ecules are packed in an efficient way (Fig. 3 ▸), as reflected in the quite high Kitaigorodskii packing index of 0.743 (Kitaigorodskii, 1965 ▸; Spek, 2009 ▸). The mean atomic volume for non-H atoms is 16.5 Å^3^ for the title compound, similar to those calculated for previously reported structures in this series (Sánchez-Lombardo *et al.*, 2014 ▸). This indicates that in this family of ionic compounds, the lattice energy can be optimized through the inclusion of a suitable number of water mol­ecules.

## Synthesis and crystallization

Good-quality single crystals of the title compound were obtained during the reaction between ammonium metavanadate (NH_4_VO_3_, 1.117 g, 9.5 mmol; Pérez-Benítez & Bernès, 2018 ▸) and metformin hydro­chloride (Metf·HCl, 0.497 g, 3 mmol; Niranjana Devi *et al.*, 2017 ▸) in 100 ml of distilled water and 6 ml of acetic acid 5% *v*/*v*. In a typical procedure, the ammonium metavanadate was dissolved by heating in a water bath and then metformin hydro­chloride was added and stirred until its dissolution. The water bath was removed and once the mixture cooled down to room temperature, the acetic acid was added. The homogeneous solution was slowly evaporated during several days at ambient conditions, which allowed the separation of reaction by-products by fractional crystallization, being the main products [H_2_Metf]_3_(V_10_O_28_)·8H_2_O and [H_2_Metf]_2_[NH_4_]_2_(V_10_O_28_)·10H_2_O (CCDC-993916 and 993917, with yields of *ca* 53 and 24%, respectively; Sánchez-Lombardo *et al.*, 2014 ▸) and the title compound, [HMetf]_2_[NH_4_]_4_(V_10_O_28_)·6H_2_O (*ca*. 5% yield). These yields are poorly reproducible, and no powder diffraction was performed on the solid phases obtained by fractional crystallization to check their purity. Therefore, we cannot rule out the presence of other crystallized compounds in this reaction.

## Refinement

Crystal data, data collection and structure refinement details are summarized in Table 2 ▸.

## Supplementary Material

Crystal structure: contains datablock(s) I. DOI: 10.1107/S2414314621006349/im4012sup1.cif

Structure factors: contains datablock(s) I. DOI: 10.1107/S2414314621006349/im4012Isup2.hkl

CCDC reference: 2090930

Additional supporting information:  crystallographic information; 3D view; checkCIF report

## Figures and Tables

**Table 1:** Hydrogen-bond geometry (Å, °)

*D*—H⋯*A*	*D*—H	H⋯*A*	*D*⋯*A*	*D*—H⋯*A*
N1—H1*A*⋯O10^i^	0.818 (19)	2.080 (19)	2.8981 (12)	178 (2)
N1—H1*B*⋯O6	0.76 (2)	2.71 (2)	3.4507 (15)	163.9 (19)
N2—H2*A*⋯O12^ii^	0.854 (18)	2.112 (18)	2.9457 (12)	165.4 (17)
N2—H2*B*⋯O15^iii^	0.849 (19)	2.072 (19)	2.9164 (16)	172.9 (17)
N4—H4*D*⋯O9^iii^	0.912 (19)	2.423 (19)	3.2993 (14)	161.1 (16)
N4—H4*E*⋯O16^iv^	0.736 (19)	2.269 (19)	2.9686 (16)	159.2 (19)
N6—H6*A*⋯O8^v^	0.882 (19)	1.865 (19)	2.7463 (13)	176.7 (17)
N6—H6*B*⋯O17^i^	0.874 (18)	1.921 (19)	2.7871 (13)	170.7 (17)
N6—H6*C*⋯O7	0.808 (19)	1.990 (19)	2.7922 (11)	172.2 (18)
N6—H6*D*⋯O2^i^	0.873 (19)	2.074 (19)	2.8541 (13)	148.3 (16)
N7—H7*A*⋯O16	0.835 (18)	2.083 (18)	2.8810 (14)	159.8 (17)
N7—H7*B*⋯O4^vi^	0.880 (18)	2.056 (18)	2.8627 (12)	152.1 (16)
N7—H7*C*⋯O1^vii^	0.843 (19)	2.072 (19)	2.9050 (12)	169.7 (17)
N7—H7*D*⋯O11^viii^	0.873 (18)	1.928 (18)	2.7957 (12)	172.1 (16)
O15—H15*A*⋯O12	0.81 (2)	2.38 (2)	3.1833 (16)	171 (2)
O15—H15*B*⋯O17^ix^	0.78 (2)	2.03 (2)	2.8046 (18)	177 (3)
O16—H16*A*⋯O15^vi^	0.80 (2)	2.05 (2)	2.8477 (18)	176 (2)
O16—H16*B*⋯O5^x^	0.83 (2)	2.23 (2)	2.8937 (13)	137 (2)
O17—H17*A*⋯O13	0.85 (1)	1.87 (2)	2.7130 (12)	172 (2)
O17—H17*B*⋯N3	0.82 (2)	2.07 (2)	2.8830 (15)	170 (2)

Symmetry codes: (i) 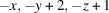 ; (ii) 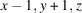 ; (iii) 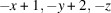 ; (iv) 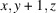 ; (v) 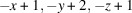 ; (vi) 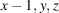 ; (vii) 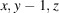 ; (viii) 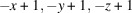 ; (ix) 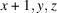 ; (x) 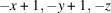 .

**Table 2:** Experimental details

Crystal data	
Chemical formula	(NH_4_)_4_(C_4_H_12_N_5_)_2_[V_10_O_28_]·6H_2_O
*M*_r_	1398.03
Crystal system, space group	Triclinic, *P* 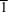
Temperature (K)	295
*a*, *b*, *c* (Å)	9.7965 (2), 10.1010 (2), 13.0974 (3)
α, β, γ (°)	81.081 (2), 70.906 (2), 63.321 (2)
*V* (Å^3^)	1094.30 (5)
*Z*	1
Radiation type	Ag *K*α, λ = 0.56083 Å
μ (mm^−1^)	1.10
Crystal size (mm)	0.26 × 0.26 × 0.19

Data collection	
Diffractometer	Stoe Stadivari
Absorption correction	Multi-scan (*X-AREA*; Stoe & Cie, 2020 ▸)
*T*_min_, *T*_max_	0.426, 0.907
No. of measured, independent and observed [*I* > 2σ(*I*)] reflections	94693, 11269, 9224
*R*_int_	0.030
(sin θ/λ)_max_ (Å^−1^)	0.851

Refinement	
*R*[*F* ^2^ > 2σ(*F* ^2^)], *wR*(*F* ^2^), *S*	0.025, 0.078, 1.06
No. of reflections	11269
No. of parameters	361
No. of restraints	9
H-atom treatment	H atoms treated by a mixture of independent and constrained refinement
Δρ_max_, Δρ_min_ (e Å^−3^)	0.47, −0.95

Computer programs: *X-AREA* (Stoe & Cie, 2020 ▸), *SHELXT2018/2* (Sheldrick, 2015*a* ▸), *SHELXL2018/3* (Sheldrick, 2015*b* ▸), *Mercury* (Macrae *et al.*, 2020 ▸) and *publCIF* (Westrip, 2010 ▸).

## full crystallographic data

### Crystal data

**Table:**

(NH_4_)_4_(C_4_H_12_N_5_)_2_[V_10_O_28_]·6H_2_O	*Z* = 1
*M**_r_* = 1398.03	*F*(000) = 700
Triclinic, *P*1	*D*_x_ = 2.121 Mg m^−^^3^
*a* = 9.7965 (2) Å	Ag *K*α radiation, λ = 0.56083 Å
*b* = 10.1010 (2) Å	Cell parameters from 131899 reflections
*c* = 13.0974 (3) Å	θ = 2.2–33.7°
α = 81.081 (2)°	µ = 1.10 mm^−^^1^
β = 70.906 (2)°	*T* = 295 K
γ = 63.321 (2)°	Tetrahedron, gold
*V* = 1094.30 (5) Å^3^	0.26 × 0.26 × 0.19 mm

### Data collection

**Table:**

Stoe Stadivari diffractometer	11269 independent reflections
Radiation source: Sealed X-ray tube, Axo Astix-f Microfocus source	9224 reflections with *I* > 2σ(*I*)
Graded multilayer mirror monochromator	*R*_int_ = 0.030
Detector resolution: 5.81 pixels mm^-1^	θ_max_ = 28.5°, θ_min_ = 2.2°
ω scans	*h* = −16→16
Absorption correction: multi-scan (X-AREA; Stoe & Cie, 2020)	*k* = −17→17
*T*_min_ = 0.426, *T*_max_ = 0.907	*l* = −22→21
94693 measured reflections	

### Refinement

**Table:**

Refinement on *F*^2^	Secondary atom site location: difference Fourier map
Least-squares matrix: full	Hydrogen site location: mixed
*R*[*F*^2^ > 2σ(*F*^2^)] = 0.025	H atoms treated by a mixture of independent and constrained refinement
*wR*(*F*^2^) = 0.078	*w* = 1/[σ^2^(*F*_o_^2^) + (0.0487*P*)^2^ + 0.0305*P*] where *P* = (*F*_o_^2^ + 2*F*_c_^2^)/3
*S* = 1.06	(Δ/σ)_max_ = 0.002
11269 reflections	Δρ_max_ = 0.47 e Å^−^^3^
361 parameters	Δρ_min_ = −0.95 e Å^−^^3^
9 restraints	Extinction correction: SHELXL-2018/3 (Sheldrick 2015b), Fc^*^=kFc[1+0.001xFc^2^λ^3^/sin(2θ)]^-1/4^
0 constraints	Extinction coefficient: 0.0048 (9)
Primary atom site location: dual	

### Special details

**Table:**

Refinement. All H atoms, with exception of the methyl groups in the HMetf^+^ cation, were refined with free coordinates and isotropic displacement parameters calculated as *U*_iso_(H) = 1.2 or 1.5×*U*_eq_(carrier atom). The geometry for the three water molecules was restrained, with target bond lengths O—H = 0.85 (2) Å and H···H separations of 1.34 (2) Å. Methyl H atoms were included using a riding model with *U*_iso_(H) = 1.5×*U*_eq_(carrier atom).

### Fractional atomic coordinates and isotropic or equivalent isotropic displacement parameters (Å^2^)

**Table:**

	*x*	*y*	*z*	*U*_iso_*/*U*_eq_	
V1	0.39655 (2)	0.84689 (2)	0.38300 (2)	0.02229 (4)	
V2	0.22257 (2)	0.64431 (2)	0.44890 (2)	0.02011 (3)	
V3	0.44412 (2)	0.63696 (2)	0.58069 (2)	0.01697 (3)	
V4	0.72567 (2)	0.57810 (2)	0.36227 (2)	0.01973 (3)	
V5	0.50248 (2)	0.59053 (2)	0.23433 (2)	0.02221 (4)	
O1	0.33595 (10)	1.02437 (8)	0.37493 (7)	0.03200 (16)	
O2	0.04039 (9)	0.66456 (10)	0.48094 (7)	0.02993 (15)	
O3	0.43043 (9)	0.62643 (8)	0.71288 (5)	0.02402 (13)	
O4	0.90793 (9)	0.55609 (9)	0.33301 (7)	0.02955 (15)	
O5	0.52329 (11)	0.57067 (11)	0.11007 (6)	0.03517 (18)	
O6	0.20204 (8)	0.83200 (8)	0.43086 (6)	0.02483 (13)	
O7	0.38555 (9)	0.81825 (7)	0.54386 (6)	0.02400 (13)	
O8	0.61636 (8)	0.77845 (7)	0.36354 (6)	0.02343 (12)	
O9	0.43593 (9)	0.79045 (8)	0.24684 (6)	0.02459 (13)	
O10	0.24430 (7)	0.63107 (7)	0.59608 (5)	0.01873 (11)	
O11	0.66939 (8)	0.57599 (7)	0.52504 (5)	0.01927 (11)	
O12	0.71111 (8)	0.55367 (8)	0.23235 (5)	0.02325 (12)	
O13	0.29624 (8)	0.60661 (8)	0.30286 (5)	0.02357 (12)	
O14	0.52088 (7)	0.40515 (7)	0.58287 (5)	0.01779 (10)	
C1	0.06586 (13)	1.15154 (12)	0.16742 (9)	0.03002 (19)	
C2	0.26523 (13)	1.04792 (11)	0.00500 (8)	0.02878 (18)	
C3	0.2491 (2)	0.8359 (2)	−0.04832 (12)	0.0567 (5)	
H3A	0.301998	0.759308	−0.002811	0.085*	
H3B	0.268909	0.794033	−0.115518	0.085*	
H3C	0.136312	0.880514	−0.012978	0.085*	
C4	0.42436 (17)	0.94509 (16)	−0.17351 (10)	0.0408 (3)	
H4A	0.377248	1.032108	−0.214239	0.061*	
H4B	0.453653	0.858445	−0.212180	0.061*	
H4C	0.517801	0.943143	−0.163104	0.061*	
N1	0.02667 (15)	1.12792 (15)	0.27388 (9)	0.0428 (3)	
H1A	−0.050 (2)	1.197 (2)	0.3093 (15)	0.051*	
H1B	0.078 (2)	1.055 (2)	0.2967 (16)	0.051*	
N2	−0.00914 (13)	1.28620 (12)	0.13003 (9)	0.0373 (2)	
H2A	−0.089 (2)	1.355 (2)	0.1705 (14)	0.045*	
H2B	0.000 (2)	1.299 (2)	0.0630 (15)	0.045*	
N3	0.17233 (12)	1.03435 (10)	0.10395 (7)	0.03244 (19)	
N4	0.32345 (14)	1.14908 (13)	−0.02036 (10)	0.0388 (2)	
H4D	0.381 (2)	1.154 (2)	−0.0901 (15)	0.047*	
H4E	0.301 (2)	1.201 (2)	0.0224 (15)	0.047*	
N5	0.30971 (13)	0.94745 (11)	−0.06891 (7)	0.03350 (19)	
N6	0.18645 (12)	1.10848 (11)	0.61533 (8)	0.02925 (17)	
H6A	0.252 (2)	1.143 (2)	0.6196 (14)	0.044*	
H6B	0.110 (2)	1.130 (2)	0.6764 (15)	0.044*	
H6C	0.238 (2)	1.022 (2)	0.5989 (14)	0.044*	
H6D	0.143 (2)	1.156 (2)	0.5644 (15)	0.044*	
N7	0.22214 (11)	0.34134 (10)	0.33362 (8)	0.02681 (15)	
H7A	0.258 (2)	0.3611 (19)	0.2689 (15)	0.040*	
H7B	0.117 (2)	0.3902 (19)	0.3535 (13)	0.040*	
H7C	0.247 (2)	0.250 (2)	0.3412 (14)	0.040*	
H7D	0.265 (2)	0.3602 (19)	0.3754 (14)	0.040*	
O15	0.99461 (17)	0.64089 (15)	0.09649 (9)	0.0543 (3)	
H15A	0.924 (2)	0.621 (3)	0.1371 (18)	0.081*	
H15B	1.021 (3)	0.683 (3)	0.1260 (19)	0.081*	
O16	0.28519 (14)	0.39198 (12)	0.10371 (8)	0.0451 (2)	
H16A	0.204 (2)	0.464 (2)	0.1036 (18)	0.068*	
H16B	0.365 (2)	0.405 (2)	0.0658 (17)	0.068*	
O17	0.07918 (11)	0.80032 (11)	0.20277 (7)	0.03818 (19)	
H17A	0.152 (2)	0.7348 (19)	0.2284 (15)	0.057*	
H17B	0.114 (2)	0.8621 (19)	0.1780 (16)	0.057*	

### Atomic displacement parameters (Å^2^)

**Table:**

	*U*^11^	*U*^22^	*U*^33^	*U*^12^	*U*^13^	*U*^23^
V1	0.02379 (7)	0.01451 (6)	0.02375 (7)	−0.00547 (5)	−0.00600 (5)	0.00227 (4)
V2	0.01597 (6)	0.02161 (7)	0.02045 (6)	−0.00631 (5)	−0.00604 (5)	0.00221 (5)
V3	0.01774 (6)	0.01375 (6)	0.01733 (6)	−0.00493 (4)	−0.00428 (4)	−0.00203 (4)
V4	0.01655 (6)	0.01890 (6)	0.02113 (6)	−0.00737 (5)	−0.00352 (5)	0.00193 (4)
V5	0.02394 (7)	0.02343 (7)	0.01623 (6)	−0.00812 (5)	−0.00540 (5)	0.00068 (5)
O1	0.0359 (4)	0.0162 (3)	0.0350 (4)	−0.0073 (3)	−0.0061 (3)	0.0027 (2)
O2	0.0196 (3)	0.0363 (4)	0.0325 (4)	−0.0114 (3)	−0.0096 (3)	0.0059 (3)
O3	0.0284 (3)	0.0222 (3)	0.0195 (3)	−0.0088 (2)	−0.0060 (2)	−0.0034 (2)
O4	0.0198 (3)	0.0333 (4)	0.0332 (4)	−0.0122 (3)	−0.0063 (3)	0.0058 (3)
O5	0.0416 (4)	0.0430 (5)	0.0189 (3)	−0.0167 (4)	−0.0084 (3)	−0.0002 (3)
O6	0.0202 (3)	0.0191 (3)	0.0278 (3)	−0.0038 (2)	−0.0057 (2)	0.0022 (2)
O7	0.0275 (3)	0.0148 (3)	0.0256 (3)	−0.0063 (2)	−0.0059 (2)	−0.0018 (2)
O8	0.0232 (3)	0.0187 (3)	0.0275 (3)	−0.0099 (2)	−0.0063 (2)	0.0027 (2)
O9	0.0254 (3)	0.0218 (3)	0.0227 (3)	−0.0078 (2)	−0.0077 (2)	0.0043 (2)
O10	0.0164 (2)	0.0165 (2)	0.0186 (2)	−0.00421 (19)	−0.00313 (19)	−0.00076 (18)
O11	0.0192 (3)	0.0188 (3)	0.0201 (3)	−0.0082 (2)	−0.0060 (2)	−0.00041 (19)
O12	0.0210 (3)	0.0247 (3)	0.0184 (3)	−0.0073 (2)	−0.0024 (2)	−0.0001 (2)
O13	0.0234 (3)	0.0266 (3)	0.0214 (3)	−0.0103 (2)	−0.0088 (2)	0.0018 (2)
O14	0.0169 (2)	0.0156 (2)	0.0186 (2)	−0.0056 (2)	−0.00443 (19)	−0.00012 (18)
C1	0.0266 (4)	0.0305 (5)	0.0278 (4)	−0.0078 (4)	−0.0042 (3)	−0.0085 (3)
C2	0.0271 (4)	0.0238 (4)	0.0266 (4)	−0.0051 (3)	−0.0037 (3)	−0.0035 (3)
C3	0.0736 (11)	0.0555 (9)	0.0407 (7)	−0.0432 (8)	0.0149 (7)	−0.0195 (6)
C4	0.0440 (6)	0.0412 (6)	0.0261 (5)	−0.0177 (5)	0.0048 (4)	−0.0050 (4)
N1	0.0390 (5)	0.0437 (6)	0.0251 (4)	−0.0017 (5)	−0.0030 (4)	−0.0092 (4)
N2	0.0353 (5)	0.0281 (4)	0.0343 (5)	−0.0034 (4)	−0.0039 (4)	−0.0079 (3)
N3	0.0342 (4)	0.0260 (4)	0.0234 (4)	−0.0058 (3)	0.0004 (3)	−0.0048 (3)
N4	0.0403 (5)	0.0346 (5)	0.0367 (5)	−0.0174 (4)	0.0002 (4)	−0.0080 (4)
N5	0.0390 (5)	0.0300 (4)	0.0237 (4)	−0.0148 (4)	0.0032 (3)	−0.0059 (3)
N6	0.0284 (4)	0.0227 (4)	0.0323 (4)	−0.0077 (3)	−0.0070 (3)	−0.0029 (3)
N7	0.0254 (4)	0.0237 (4)	0.0315 (4)	−0.0102 (3)	−0.0087 (3)	−0.0008 (3)
O15	0.0633 (7)	0.0591 (7)	0.0337 (5)	−0.0251 (6)	−0.0099 (5)	0.0053 (4)
O16	0.0503 (6)	0.0430 (5)	0.0348 (4)	−0.0206 (4)	−0.0042 (4)	0.0031 (4)
O17	0.0328 (4)	0.0406 (5)	0.0318 (4)	−0.0107 (4)	−0.0091 (3)	0.0082 (3)

### Geometric parameters (Å, º)

**Table:**

V1—O1	1.6137 (7)	V5—O14^i^	2.3350 (6)
V1—O9	1.8226 (7)	C1—N2	1.3271 (16)
V1—O6	1.8689 (8)	C1—N1	1.3332 (15)
V1—O8	1.8811 (7)	C1—N3	1.3451 (13)
V1—O7	2.0554 (7)	C2—N4	1.3318 (16)
V1—O14^i^	2.3173 (6)	C2—N5	1.3374 (13)
V1—V5	3.0663 (2)	C2—N3	1.3460 (14)
V1—V2	3.0755 (2)	C3—N5	1.4474 (18)
V1—V3	3.1011 (2)	C3—H3A	0.9600
V1—V4	3.1042 (2)	C3—H3B	0.9600
V2—O2	1.6161 (8)	C3—H3C	0.9600
V2—O6	1.8001 (7)	C4—N5	1.4554 (14)
V2—O13	1.8440 (7)	C4—H4A	0.9600
V2—O10	1.9854 (7)	C4—H4B	0.9600
V2—O11^i^	2.0185 (7)	C4—H4C	0.9600
V2—O14^i^	2.2343 (6)	N1—H1A	0.818 (19)
V2—V4^i^	3.0815 (2)	N1—H1B	0.76 (2)
V3—O3	1.6825 (7)	N2—H2A	0.854 (18)
V3—O7	1.6968 (7)	N2—H2B	0.849 (19)
V3—O11	1.9098 (7)	N4—H4D	0.912 (19)
V3—O10	1.9284 (7)	N4—H4E	0.736 (19)
V3—O14	2.1121 (6)	N6—H6A	0.882 (19)
V3—O14^i^	2.1349 (6)	N6—H6B	0.874 (18)
V3—V5^i^	3.0737 (2)	N6—H6C	0.808 (19)
V4—O4	1.6145 (7)	N6—H6D	0.873 (19)
V4—O8	1.8153 (7)	N7—H7A	0.835 (18)
V4—O12	1.8158 (7)	N7—H7B	0.880 (18)
V4—O10^i^	2.0094 (7)	N7—H7C	0.843 (19)
V4—O11	2.0192 (6)	N7—H7D	0.873 (18)
V4—O14^i^	2.2168 (6)	O15—H15A	0.812 (16)
V4—V5	3.1128 (2)	O15—H15B	0.778 (16)
V5—O5	1.6055 (8)	O16—H16A	0.804 (15)
V5—O9	1.8385 (7)	O16—H16B	0.830 (15)
V5—O13	1.8649 (7)	O17—H17A	0.850 (14)
V5—O12	1.8978 (7)	O17—H17B	0.820 (15)
V5—O3^i^	2.0607 (7)		
			
O1—V1—O9	104.84 (4)	O12—V4—V1	84.23 (2)
O1—V1—O6	101.04 (4)	O10^i^—V4—V1	124.447 (19)
O9—V1—O6	92.16 (3)	O11—V4—V1	87.655 (19)
O1—V1—O8	102.51 (4)	O14^i^—V4—V1	48.162 (16)
O9—V1—O8	91.10 (3)	V2^i^—V4—V1	120.184 (6)
O6—V1—O8	154.52 (3)	O4—V4—V5	136.16 (3)
O1—V1—O7	98.71 (4)	O8—V4—V5	84.21 (2)
O9—V1—O7	156.44 (3)	O12—V4—V5	33.88 (2)
O6—V1—O7	83.85 (3)	O10^i^—V4—V5	87.47 (2)
O8—V1—O7	83.12 (3)	O11—V4—V5	124.45 (2)
O1—V1—O14^i^	172.87 (4)	O14^i^—V4—V5	48.462 (16)
O9—V1—O14^i^	82.28 (3)	V2^i^—V4—V5	119.451 (6)
O6—V1—O14^i^	78.35 (3)	V1—V4—V5	59.105 (6)
O8—V1—O14^i^	77.07 (3)	O5—V5—O9	104.08 (4)
O7—V1—O14^i^	74.17 (2)	O5—V5—O13	102.38 (4)
O1—V1—V5	138.11 (3)	O9—V5—O13	91.70 (3)
O9—V1—V5	33.28 (2)	O5—V5—O12	102.89 (4)
O6—V1—V5	84.42 (2)	O9—V5—O12	90.47 (3)
O8—V1—V5	84.55 (2)	O13—V5—O12	153.29 (3)
O7—V1—V5	123.18 (2)	O5—V5—O3^i^	100.16 (4)
O14^i^—V1—V5	49.017 (16)	O9—V5—O3^i^	155.75 (3)
O1—V1—V2	133.39 (3)	O13—V5—O3^i^	83.71 (3)
O9—V1—V2	83.40 (3)	O12—V5—O3^i^	83.45 (3)
O6—V1—V2	32.35 (2)	O5—V5—O14^i^	174.46 (4)
O8—V1—V2	123.42 (2)	O9—V5—O14^i^	81.46 (3)
O7—V1—V2	80.88 (2)	O13—V5—O14^i^	77.01 (3)
O14^i^—V1—V2	46.362 (16)	O12—V5—O14^i^	77.00 (3)
V5—V1—V2	61.310 (6)	O3^i^—V5—O14^i^	74.30 (2)
O1—V1—V3	129.40 (3)	O5—V5—V1	137.01 (4)
O9—V1—V3	125.76 (2)	O9—V5—V1	32.96 (2)
O6—V1—V3	79.55 (2)	O13—V5—V1	83.39 (2)
O8—V1—V3	78.07 (2)	O12—V5—V1	84.06 (2)
O7—V1—V3	30.690 (19)	O3^i^—V5—V1	122.825 (19)
O14^i^—V1—V3	43.479 (15)	O14^i^—V5—V1	48.521 (15)
V5—V1—V3	92.496 (6)	O5—V5—V3^i^	131.12 (4)
V2—V1—V3	61.458 (5)	O9—V5—V3^i^	124.81 (2)
O1—V1—V4	134.70 (3)	O13—V5—V3^i^	78.32 (2)
O9—V1—V4	81.46 (2)	O12—V5—V3^i^	78.62 (2)
O6—V1—V4	123.80 (2)	O3^i^—V5—V3^i^	30.960 (18)
O8—V1—V4	32.23 (2)	O14^i^—V5—V3^i^	43.346 (15)
O7—V1—V4	81.57 (2)	V1—V5—V3^i^	91.865 (6)
O14^i^—V1—V4	45.455 (15)	O5—V5—V4	135.05 (4)
V5—V1—V4	60.588 (6)	O9—V5—V4	80.99 (2)
V2—V1—V4	91.644 (6)	O13—V5—V4	122.29 (2)
V3—V1—V4	61.402 (5)	O12—V5—V4	32.23 (2)
O2—V2—O6	103.19 (4)	O3^i^—V5—V4	81.44 (2)
O2—V2—O13	102.93 (4)	O14^i^—V5—V4	45.285 (16)
O6—V2—O13	94.22 (3)	V1—V5—V4	60.308 (5)
O2—V2—O10	99.24 (4)	V3^i^—V5—V4	61.383 (5)
O6—V2—O10	92.09 (3)	V3—O3—V5^i^	109.98 (3)
O13—V2—O10	154.87 (3)	V2—O6—V1	113.89 (4)
O2—V2—O11^i^	98.97 (4)	V3—O7—V1	111.12 (3)
O6—V2—O11^i^	156.53 (3)	V4—O8—V1	114.22 (4)
O13—V2—O11^i^	88.24 (3)	V1—O9—V5	113.76 (4)
O10—V2—O11^i^	76.68 (3)	V3—O10—V2	107.49 (3)
O2—V2—O14^i^	173.66 (3)	V3—O10—V4^i^	106.61 (3)
O6—V2—O14^i^	82.01 (3)	V2—O10—V4^i^	100.95 (3)
O13—V2—O14^i^	80.07 (3)	V3—O11—V2^i^	107.71 (3)
O10—V2—O14^i^	76.76 (3)	V3—O11—V4	107.45 (3)
O11^i^—V2—O14^i^	75.42 (3)	V2^i^—O11—V4	99.49 (3)
O2—V2—V1	136.83 (3)	V4—O12—V5	113.89 (3)
O6—V2—V1	33.75 (2)	V2—O13—V5	115.19 (4)
O13—V2—V1	83.44 (2)	V3—O14—V3^i^	102.04 (3)
O10—V2—V1	88.47 (2)	V3—O14—V4^i^	93.65 (2)
O11^i^—V2—V1	124.06 (2)	V3^i^—O14—V4^i^	93.42 (2)
O14^i^—V2—V1	48.643 (16)	V3—O14—V2^i^	93.73 (2)
O2—V2—V4^i^	89.10 (3)	V3^i^—O14—V2^i^	92.47 (2)
O6—V2—V4^i^	131.89 (3)	V4^i^—O14—V2^i^	169.39 (3)
O13—V2—V4^i^	128.50 (2)	V3—O14—V1^i^	169.74 (3)
O10—V2—V4^i^	39.806 (19)	V3^i^—O14—V1^i^	88.20 (2)
O11^i^—V2—V4^i^	40.263 (18)	V4^i^—O14—V1^i^	86.38 (2)
O14^i^—V2—V4^i^	84.694 (17)	V2^i^—O14—V1^i^	84.99 (2)
V1—V2—V4^i^	120.428 (6)	V3—O14—V5^i^	87.30 (2)
O3—V3—O7	107.11 (4)	V3^i^—O14—V5^i^	170.66 (3)
O3—V3—O11	97.73 (3)	V4^i^—O14—V5^i^	86.25 (2)
O7—V3—O11	97.83 (3)	V2^i^—O14—V5^i^	86.50 (2)
O3—V3—O10	97.47 (3)	V1^i^—O14—V5^i^	82.46 (2)
O7—V3—O10	96.04 (3)	N2—C1—N1	118.43 (10)
O11—V3—O10	155.39 (3)	N2—C1—N3	123.86 (10)
O3—V3—O14	88.41 (3)	N1—C1—N3	117.57 (11)
O7—V3—O14	164.44 (3)	N4—C2—N5	118.72 (10)
O11—V3—O14	80.64 (3)	N4—C2—N3	123.49 (10)
O10—V3—O14	80.55 (3)	N5—C2—N3	117.62 (10)
O3—V3—O14^i^	166.38 (3)	N5—C3—H3A	109.5
O7—V3—O14^i^	86.51 (3)	N5—C3—H3B	109.5
O11—V3—O14^i^	80.29 (3)	H3A—C3—H3B	109.5
O10—V3—O14^i^	80.39 (3)	N5—C3—H3C	109.5
O14—V3—O14^i^	77.96 (3)	H3A—C3—H3C	109.5
O3—V3—V5^i^	39.06 (2)	H3B—C3—H3C	109.5
O7—V3—V5^i^	146.17 (2)	N5—C4—H4A	109.5
O11—V3—V5^i^	89.66 (2)	N5—C4—H4B	109.5
O10—V3—V5^i^	90.04 (2)	H4A—C4—H4B	109.5
O14—V3—V5^i^	49.358 (17)	N5—C4—H4C	109.5
O14^i^—V3—V5^i^	127.319 (17)	H4A—C4—H4C	109.5
O3—V3—V1	145.30 (3)	H4B—C4—H4C	109.5
O7—V3—V1	38.19 (2)	C1—N1—H1A	115.0 (14)
O11—V3—V1	89.69 (2)	C1—N1—H1B	119.0 (15)
O10—V3—V1	88.75 (2)	H1A—N1—H1B	126 (2)
O14—V3—V1	126.279 (18)	C1—N2—H2A	122.6 (12)
O14^i^—V3—V1	48.321 (17)	C1—N2—H2B	119.7 (12)
V5^i^—V3—V1	175.629 (6)	H2A—N2—H2B	114.9 (17)
O4—V4—O8	102.13 (4)	C1—N3—C2	122.59 (10)
O4—V4—O12	102.49 (4)	C2—N4—H4D	118.1 (12)
O8—V4—O12	95.66 (3)	C2—N4—H4E	117.1 (15)
O4—V4—O10^i^	99.74 (4)	H4D—N4—H4E	124.7 (19)
O8—V4—O10^i^	155.62 (3)	C2—N5—C3	122.03 (10)
O12—V4—O10^i^	89.99 (3)	C2—N5—C4	120.63 (11)
O4—V4—O11	99.12 (4)	C3—N5—C4	117.34 (10)
O8—V4—O11	89.83 (3)	H6A—N6—H6B	106.4 (16)
O12—V4—O11	156.02 (3)	H6A—N6—H6C	108.3 (17)
O10^i^—V4—O11	76.13 (3)	H6B—N6—H6C	117.5 (17)
O4—V4—O14^i^	174.28 (3)	H6A—N6—H6D	109.4 (17)
O8—V4—O14^i^	81.05 (3)	H6B—N6—H6D	107.1 (16)
O12—V4—O14^i^	81.78 (3)	H6C—N6—H6D	108.0 (17)
O10^i^—V4—O14^i^	76.30 (2)	H7A—N7—H7B	109.0 (16)
O11—V4—O14^i^	76.03 (3)	H7A—N7—H7C	109.8 (16)
O4—V4—V2^i^	89.60 (3)	H7B—N7—H7C	109.4 (16)
O8—V4—V2^i^	130.07 (2)	H7A—N7—H7D	112.0 (16)
O12—V4—V2^i^	129.23 (2)	H7B—N7—H7D	111.8 (15)
O10^i^—V4—V2^i^	39.240 (19)	H7C—N7—H7D	104.8 (16)
O11—V4—V2^i^	40.246 (19)	H15A—O15—H15B	112 (2)
O14^i^—V4—V2^i^	84.733 (17)	H16A—O16—H16B	112 (2)
O4—V4—V1	135.49 (3)	H17A—O17—H17B	102.5 (18)
O8—V4—V1	33.55 (2)		
			
O7—V3—O3—V5^i^	179.48 (4)	V3—V1—O9—V5	2.25 (5)
O11—V3—O3—V5^i^	−79.82 (4)	V4—V1—O9—V5	47.45 (3)
O10—V3—O3—V5^i^	80.77 (4)	O5—V5—O9—V1	178.29 (5)
O14—V3—O3—V5^i^	0.51 (4)	O13—V5—O9—V1	75.08 (4)
O14^i^—V3—O3—V5^i^	0.80 (16)	O12—V5—O9—V1	−78.29 (4)
V1—V3—O3—V5^i^	179.443 (13)	O3^i^—V5—O9—V1	−3.32 (11)
O2—V2—O6—V1	−175.89 (4)	O14^i^—V5—O9—V1	−1.51 (4)
O13—V2—O6—V1	−71.55 (4)	V3^i^—V5—O9—V1	−1.85 (5)
O10—V2—O6—V1	84.11 (4)	V4—V5—O9—V1	−47.36 (3)
O11^i^—V2—O6—V1	23.77 (10)	O4—V4—O12—V5	−174.54 (4)
O14^i^—V2—O6—V1	7.80 (4)	O8—V4—O12—V5	−70.76 (4)
V4^i^—V2—O6—V1	83.24 (4)	O10^i^—V4—O12—V5	85.48 (4)
O1—V1—O6—V2	179.64 (4)	O11—V4—O12—V5	31.67 (9)
O9—V1—O6—V2	74.06 (4)	O14^i^—V4—O12—V5	9.32 (4)
O8—V1—O6—V2	−23.07 (10)	V2^i^—V4—O12—V5	85.57 (4)
O7—V1—O6—V2	−82.65 (4)	V1—V4—O12—V5	−39.17 (3)
O14^i^—V1—O6—V2	−7.60 (4)	O5—V5—O12—V4	176.70 (5)
V5—V1—O6—V2	41.65 (4)	O9—V5—O12—V4	72.14 (4)
V3—V1—O6—V2	−51.93 (3)	O13—V5—O12—V4	−22.59 (9)
V4—V1—O6—V2	−7.11 (5)	O3^i^—V5—O12—V4	−84.33 (4)
O3—V3—O7—V1	−179.96 (4)	O14^i^—V5—O12—V4	−8.99 (3)
O11—V3—O7—V1	79.41 (4)	V1—V5—O12—V4	39.76 (3)
O10—V3—O7—V1	−80.21 (4)	V3^i^—V5—O12—V4	−53.35 (3)
O14—V3—O7—V1	−3.81 (14)	O2—V2—O13—V5	175.65 (4)
O14^i^—V3—O7—V1	−0.27 (4)	O6—V2—O13—V5	71.08 (4)
V5^i^—V3—O7—V1	−179.378 (13)	O10—V2—O13—V5	−33.02 (10)
O4—V4—O8—V1	174.69 (4)	O11^i^—V2—O13—V5	−85.55 (4)
O12—V4—O8—V1	70.60 (4)	O14^i^—V2—O13—V5	−10.04 (4)
O10^i^—V4—O8—V1	−32.00 (10)	V1—V2—O13—V5	39.04 (3)
O11—V4—O8—V1	−86.02 (4)	V4^i^—V2—O13—V5	−85.02 (4)
O14^i^—V4—O8—V1	−10.14 (4)	O5—V5—O13—V2	−175.94 (5)
V2^i^—V4—O8—V1	−85.43 (4)	O9—V5—O13—V2	−71.13 (4)
V5—V4—O8—V1	38.66 (3)	O12—V5—O13—V2	23.31 (10)
O1—V1—O8—V4	−177.47 (4)	O3^i^—V5—O13—V2	85.00 (4)
O9—V1—O8—V4	−72.00 (4)	O14^i^—V5—O13—V2	9.71 (4)
O6—V1—O8—V4	25.37 (10)	V1—V5—O13—V2	−39.18 (3)
O7—V1—O8—V4	85.10 (4)	V3^i^—V5—O13—V2	54.11 (3)
O14^i^—V1—O8—V4	9.83 (3)	V4—V5—O13—V2	9.28 (5)
V5—V1—O8—V4	−39.33 (3)	N2—C1—N3—C2	30.11 (19)
V2—V1—O8—V4	10.82 (5)	N1—C1—N3—C2	−154.27 (13)
V3—V1—O8—V4	54.39 (3)	N4—C2—N3—C1	34.71 (19)
O1—V1—O9—V5	−178.43 (4)	N5—C2—N3—C1	−150.09 (12)
O6—V1—O9—V5	−76.42 (4)	N4—C2—N5—C3	−178.69 (15)
O8—V1—O9—V5	78.31 (4)	N3—C2—N5—C3	5.88 (19)
O7—V1—O9—V5	3.13 (11)	N4—C2—N5—C4	2.39 (18)
O14^i^—V1—O9—V5	1.52 (4)	N3—C2—N5—C4	−173.04 (12)
V2—V1—O9—V5	−45.22 (4)		

Symmetry code: (i) −*x*+1, −*y*+1, −*z*+1.

### Hydrogen-bond geometry (Å, º)

**Table:**

*D*—H···*A*	*D*—H	H···*A*	*D*···*A*	*D*—H···*A*
N1—H1*A*···O10^ii^	0.818 (19)	2.080 (19)	2.8981 (12)	178 (2)
N1—H1*B*···O6	0.76 (2)	2.71 (2)	3.4507 (15)	163.9 (19)
N2—H2*A*···O12^iii^	0.854 (18)	2.112 (18)	2.9457 (12)	165.4 (17)
N2—H2*B*···O15^iv^	0.849 (19)	2.072 (19)	2.9164 (16)	172.9 (17)
N4—H4*D*···O9^iv^	0.912 (19)	2.423 (19)	3.2993 (14)	161.1 (16)
N4—H4*E*···O16^v^	0.736 (19)	2.269 (19)	2.9686 (16)	159.2 (19)
N6—H6*A*···O8^vi^	0.882 (19)	1.865 (19)	2.7463 (13)	176.7 (17)
N6—H6*B*···O17^ii^	0.874 (18)	1.921 (19)	2.7871 (13)	170.7 (17)
N6—H6*C*···O7	0.808 (19)	1.990 (19)	2.7922 (11)	172.2 (18)
N6—H6*D*···O2^ii^	0.873 (19)	2.074 (19)	2.8541 (13)	148.3 (16)
N7—H7*A*···O16	0.835 (18)	2.083 (18)	2.8810 (14)	159.8 (17)
N7—H7*B*···O2^vii^	0.880 (18)	2.367 (17)	2.9194 (12)	121.1 (14)
N7—H7*B*···O4^viii^	0.880 (18)	2.056 (18)	2.8627 (12)	152.1 (16)
N7—H7*C*···O1^ix^	0.843 (19)	2.072 (19)	2.9050 (12)	169.7 (17)
N7—H7*D*···O11^i^	0.873 (18)	1.928 (18)	2.7957 (12)	172.1 (16)
O15—H15*A*···O4	0.81 (2)	2.52 (2)	3.0266 (14)	122 (2)
O15—H15*A*···O12	0.81 (2)	2.38 (2)	3.1833 (16)	171 (2)
O15—H15*B*···O17^x^	0.78 (2)	2.03 (2)	2.8046 (18)	177 (3)
O16—H16*A*···O15^viii^	0.80 (2)	2.05 (2)	2.8477 (18)	176 (2)
O16—H16*B*···O5^xi^	0.83 (2)	2.23 (2)	2.8937 (13)	137 (2)
O17—H17*A*···O13	0.85 (1)	1.87 (2)	2.7130 (12)	172 (2)
O17—H17*B*···N3	0.82 (2)	2.07 (2)	2.8830 (15)	170 (2)

Symmetry codes: (i) −*x*+1, −*y*+1, −*z*+1; (ii) −*x*, −*y*+2, −*z*+1; (iii) *x*−1, *y*+1, *z*; (iv) −*x*+1, −*y*+2, −*z*; (v) *x*, *y*+1, *z*; (vi) −*x*+1, −*y*+2, −*z*+1; (vii) −*x*, −*y*+1, −*z*+1; (viii) *x*−1, *y*, *z*; (ix) *x*, *y*−1, *z*; (x) *x*+1, *y*, *z*; (xi) −*x*+1, −*y*+1, −*z*.
